# From wartime loudspeakers to digital networks: communist persuasion and pandemic politics in Vietnam

**DOI:** 10.1177/1329878X20953226

**Published:** 2020-11

**Authors:** Giang Nguyen-Thu

**Affiliations:** The University of Queensland, Australia

**Keywords:** Asian digital transaction, COVID-19, late-socialism, political legitimacy, Vietnamese digital culture

## Abstract

This commentary discusses the overall propaganda win of the Vietnamese government for its COVID-19 success and reveals the underlying logics of communist legitimacy in the wake of digital and biological virality.

## Digital transactions in pandemic late-socialism

On 8 June 2020, Vietnamese prime minister Nguyễn Xuân Phúc allegedly remarked on the country’s victory over COVID-19 by comparing the great achievements of Vietnam today, among which the pandemic success was just an example, with the impoverished years after the end of the Vietnam War in 1975. He also levelled Vietnam’s success against the general failure of the West, particularly the United States, in controlling the virus. At one point, he declared that ‘if the utility poles in the US had legs, they would now be going to Vietnam’. His statement was soon reported online by mainstream media but quickly removed after the story went unfavourably viral on Facebook, where many dissidents mocked Mr Phúc for his delusional and politically insensitive comparison. By recycling the metaphor of ‘utility poles with legs’, which was commonly uttered in secrecy during the 1980s to describe tragic waves of people escaping punishing communism after the fall of Saigon, Mr Phúc’s statement rubbed salt to the open wound of North-South division, and as such, failed to achieve his goal of using the COVID-19 victory to claim more political legitimacy. Within a few hours, his saying was nowhere to be found in all state-run channels, but public critiques continue to linger on Facebook, YouTube and dissident blogs – all are relatively new digital platforms over which the government has no immediate control.

Mr Phúc’s statement was pronounced in the context of Vietnam safely returning to its previous ‘normal’ after nearly 2 months without any detected community transmission of the novel coronavirus. Such an achievement was dubbed ‘the envy of the world’ by *ABC News* (Walden, 2020) and allowed Vietnam to enter *Forbes*’ top 20 list of ‘safest’ places in the pandemic (Koetsier, 2020). Mr Phúc’s statement, in its most explicit level, thus provides a rather sensible pairing between Vietnam’s exemplary success and the United States’ combination of shocking pandemic casualty and massive national protests. While his statement was received with more resistance than approval, even the most hostile critic failed to deny the government’s COVID-19 victory. As long as the virus lingers, which is to be expected, it continues to give the State a reason, albeit under intense pressure, to sustain and legitimize communist leadership under the banner of pandemic management. But the fact that the prime minister could not wait to use the COVID-19 to wash away historical trauma caused by the communist regime also indicates that the sense of social hope and informational transparency briefly experienced during the pandemic period is unlikely to be linked with any improvement in political reformation.

The handling of Mr Phúc’s statement reveals the typical scenario of Vietnamese media censorship in the wake of the digital age (Nguyen-Thu, 2018), where one sees less harsh punishment than constant negotiations, or the dynamics of what Athique (2019) terms ‘digital transactions’ between state-run mass media and the relatively ‘free’ social media. What is particular about the (de)circulation of Mr Phúc’s statement is that the pandemic situation allows us to see more clearly how the Party-State actively adopts new communication technologies in promoting its political legitimacy, while at the same time struggles to regulate and minimize the digital affordances that have greatly expanded Vietnamese citizens’ capacity to participate, co-produce and destabilize public discourses.

## Weaving pandemic solidarity in a post-war society

The mentioning of the utility poles by Mr Phúc prompts us to revisit Vietnam’s war history as an entry point to understand COVID-19 communication practices. A utility pole in Vietnam is usually attached to a loudspeaker that blares propagandist messages to the surrounding neighbourhood at 6:30 a.m. and 4:30 p.m. daily. Now blamed as a source of urban annoyance and a symbol of ideological conservatism (Võ, 2017), this loudspeaker system, which was first used in the 1960s to deliver warnings of American bombing, continues to serve the nation in the current ‘war against COVID-19’. In this new battlefield, however, the combination of the obsolete loudspeaker system with up-to-minute Facebook posting and smartphone messaging indicates how the communist leaders are willing to engage with new digital technologies while still insisting on maintaining their conservative legacy.

During the most intense pandemic period from March to May 2020, for the first time in many decades Vietnamese publics had experienced something close to ‘transparency’. It was quite clear that the State understood the impossibility of concealing outbreaks by learning from the tragedy in Wuhan and the Vietnamese experience with SARS in 2002. Instead of hiding bad news as normally seen in communist propaganda, concerted efforts were made to (over-)alert the publics about the severity of the situation. There was massive involvement of old and new communication channels, from loudspeakers, neighbourhood watching, door-to-door warning, to constant updates directly sent to smartphones and through popular messaging apps, such as Zalo and Viber. On Facebook, the official page by the government attracted unprecedented popularity for its highly engaging posts, sometimes at the frequency of 10 feeds a day. These posts quickly clarified groundless rumour by sharing newest data, photos and videos. Some posts attracted a dozen thousand of reactions and hundreds of (filtered) comments. During the most vulnerable days in late March and early April when community transmission seemed to get out of control, the Party-State had managed to deliver the message of hope and order that most citizens were happy to follow.

The wartime metaphor also demonstrates how the virus serves as a newly found enemy that can unite the State and its citizens, at least for a few months. Being a non-human foe with so much threatening urgency and so little history of political complexity, the virus transcends political tension often associated with other ‘enemies’, such as the United States in terms of ideological opposition and China in terms of territorial disputes. The utility pole, whether as a symbol of divisive politics or a physical host of communist propaganda, reminds us that the pandemic’s globalizing force is never separated from the cultural and political baggage that each community carries.

The propagandist efforts by the Vietnamese state, however, should be located in the context of strong social responsibilities and community bonding in a country where the memories of brutal wars and extreme poverty are still vivid at the grassroots level. On the one hand, the society’s post-traumatic desire to maintain basic security is well accounted for by the State. On the other hand, the ‘obedience’ from below is less an act of passive acceptance than a collective choice in protecting a shared future. The strict and early application of contact tracing, border closing, forced quarantine, social distancing and mask wearing were all widely considered common-sense measures to protect the community. In mid-April 2020, while there had been only 270 cases of infection, Vietnam had put 60,000 people under compulsory quarantine (Nhật, 2020), not to mention a massive number of people under home isolation and neighbourhood lockdown. Instead of staying in five-star hotels as in Australia, people in Vietnam suffered their 14-day quarantine in military-run facilities or university dormitories with literally nothing but a bunk bed and a shared toilet. Such poor conditions, however, received near-zero public complaints. On the contrary, any pronounced grievance was met with waves of public criticism from social networks, often in a nationalist tone (Mai, 2020).

Here, it is worth noting that Vietnamese audiences are never docile subjects of communist propaganda. Media consumers in Vietnam master the arts of reading between the lines and enjoy playing with official messages (Harms, 2016: 109–110). These practices of tactical resistance have become ever more prominent at the advent of digital networking when more than 60 million Vietnamese citizens own a Facebook account, of which the State can only censor ‘from afar’ through back-and-forth negotiations with Facebook (Pearson, 2020). The strong presence of social solidarity and the sustenance of hope throughout the pandemics thus reveal rather surprising synchronization among neighbourhood webs of rumour, digital networks of citizen journalism and top-down media feeds. Informational transparency, social solidarity and a strong sense of collective responsibility – whether in the context of semi-authoritarianism or liberal democracy – prove to be the best combination to cope with contagious vulnerabilities in pandemic times.

## Conclusion: communist persuasion and the logic of non-alternative

The legitimacy of communist leadership, which is set against disastrous COVID-19 situations across the globe, gradually leads to a relatively defeating bargain between everyday ‘normality’ and structural political reform. These two options should never be mutually exclusive but have increasingly become so, at least if the State continues to keep the serious threat of coronavirus at bay. In the face of an undeniable Western failure, the choice of everyday normality under (and *despite*) the communist regime has earned significant credits in the Vietnamese public sphere. The rhetoric of ‘no better alternative’ becomes much more persuasive when the ideal of liberal democracy, which has long inspired Vietnamese oppositional politics (Harris and Perlez, 2016), is losing its appeal at the brutal surfacing of racialized inhumanities and medical failure in the United States. Although the Vietnamese publics are never naïve in putting uncritical trust in the communist leaders, as seen in the viral resistance against Mr Phúc’s reference to the ‘running utility poles’, the COVID-19 situation can still be considered a propaganda win on the side of the regime. Such a win, however, operates more in the form of a practical and temporary alliance against a newly found enemy than a matter of systemic ideological docility.

Social and cultural spaces of public feelings in Vietnam, which range from digital networking (Phuong, 2017), popular music (Hoang, 2020) to popular television (Nguyen-Thu, 2019), have long served as vital sites of negotiation and contestation between top-down and bottom-up agendas. In these ‘grey’ areas, the State often tolerates soft-toned critiques to allow the venting out of latent public grievance. The Vietnamese case of COVID-19 indicates that in the specific conjuncture of intense global insecurity and local desires for social order, the space of popular culture can lean to the side of top-down power. What remains to be seen is how the State manages to sustain its hard-earned legitimacy in the face of the constant threat of coronavirus resurgence and a gloomy post-pandemic economic recession. Another issue is how Vietnamese oppositional politics finds its way to transcend the rhetoric of ‘non-alternative’ by reaching beyond the self-trapping binary between the Western ideal of liberal democracy and the pandemic-induced persuasion from above.
